# Deep learning captures the effect of epistasis in multifactorial diseases

**DOI:** 10.3389/fmed.2024.1479717

**Published:** 2025-01-07

**Authors:** Vladislav Perelygin, Alexey Kamelin, Nikita Syzrantsev, Layal Shaheen, Anna Kim, Nikolay Plotnikov, Anna Ilinskaya, Valery Ilinsky, Alexander Rakitko, Maria Poptsova

**Affiliations:** ^1^International Laboratory of Bioinformatics, AI and Digital Sciences Institute, Faculty of Computer Science, HSE University, Moscow, Russia; ^2^Genotek Ltd., Moscow, Russia; ^3^Phystech School of Biological and Medical Physics, Moscow Institute of Physics and Technology, Moscow, Russia; ^4^Eligens SIA, Mārupe, Latvia

**Keywords:** polygenic risk score, multifactorial diseases, epistasis, obesity, type 1 diabetes, psoriasis, machine learning, deep learning

## Abstract

**Background:**

Polygenic risk score (PRS) prediction is widely used to assess the risk of diagnosis and progression of many diseases. Routinely, the weights of individual SNPs are estimated by the linear regression model that assumes independent and linear contribution of each SNP to the phenotype. However, for complex multifactorial diseases such as Alzheimer’s disease, diabetes, cardiovascular disease, cancer, and others, association between individual SNPs and disease could be non-linear due to epistatic interactions. The aim of the presented study is to explore the power of non-linear machine learning algorithms and deep learning models to predict the risk of multifactorial diseases with epistasis.

**Methods:**

Simulated data with 2- and 3-loci interactions and tested three different models of epistasis: additive, multiplicative and threshold, were generated using the GAMETES. Penetrance tables were generated using PyTOXO package. For machine learning methods we used multilayer perceptron (MLP), convolutional neural network (CNN) and recurrent neural network (RNN), Lasso regression, random forest and gradient boosting models. Performance of machine learning models were assessed using accuracy, AUC-ROC, AUC-PR, recall, precision, and F1 score.

**Results:**

First, we tested ensemble tree methods and deep learning neural networks against LASSO linear regression model on simulated data with different types and strength of epistasis. The results showed that with the increase of strength of epistasis effect, non-linear models significantly outperform linear. Then the higher performance of non-linear models over linear was confirmed on real genetic data for multifactorial phenotypes such as obesity, type 1 diabetes, and psoriasis. From non-linear models, gradient boosting appeared to be the best model in obesity and psoriasis while deep learning methods significantly outperform linear approaches in type 1 diabetes.

**Conclusion:**

Overall, our study underscores the efficacy of non-linear models and deep learning approaches in more accurately accounting for the effects of epistasis in simulations with specific configurations and in the context of certain diseases.

## 1 Introduction

Modern technologies have enabled the use of genomic data to predict and customize strategies for preventing and treating diseases. Millions of single-nucleotide polymorphisms (SNPs) exist in the human genome, and genome-wide association studies (GWAS) help to identify associative links between SNPs and various diseases (1). Frequently polymorphisms with weak individual effects may collectively exhibit a strong correlation with a disease (2). Polygenic Risk Score (PRS), a linear regression model that uses individual SNPs with weights derived from GWAS, has traditionally been used to assess the risk of multifactorial disease manifestation. Although PRS has rightfully become the most popular tool due to its simplicity and good predictive ability, it has significant limitations, such as inability to account for non-linear effect of epistasis. Although, historically this term has been used to describe various genetic events, the most suitable definition was proposed by Fisher (3). That is statistical epistasis, and it refers to a phenomenon where the effect of genetic variants on disease is non-additive. Epistasis is a field of active study, and it has already been proven to have a significant effect in a number of diseases (4). Epistasis is a challenging aspect in building a reliable polygenic risk model, as linear approaches are often insufficient to capture non-linear relationships between genetic variants and disease.

Machine learning techniques may help to overcome some of PRS limitations. For instance, deep neural networks (DNN) have improved PRS for predicting breast cancer (5). DNN demonstrated better results (AUC ROC 0.674) than any other approach, including best linear unbiased estimator (AUC ROC 0.642), BayesA (AUC ROC 0.645), LDpred (AUC ROC 0.624), random forest (AUC ROC 0.636) and gradient boosting (AUC ROC 0.651). The same conclusion was reached by the researchers also for the breast cancer and breast cancer subtypes in Chinese population in the work (6), although the difference in performance was less significant (AUC ROC of 0.601 for DNN and 0.598 for logistic ridge regression). Neural network-based approach has also proven effective in predicting other risks, including some heart conditions (myocardial infarction, stroke and others) (7), Alzheimer’s disease (8, 9) and 10 phenotypes from UK biobank (10). In another study based on UK biobank phenotypes, authors showed that gradient boosting modes outperform linear when considering non-genetic covariates (11). In this study, we evaluated the potential of various machine-learning methods on simulated data with epistasis. After that, we tested the performance of these models on multifactorial diseases: obesity, type 1 diabetes, and psoriasis.

Obesity is a global health problem that has raised major concerns in recent decades. According to the World Health Organization (WHO), obesity rates have nearly tripled worldwide since 1975, with over 650 million adults categorized as obese (12). Obesity is associated with numerous health risks and chronic conditions, including type 2 diabetes, cardiovascular disease, high blood pressure, some types of cancer, and respiratory problems (13). In addition to that, obesity has also a significant impact on a person’s mental well-being, leading to anxiety and depression (14). The causes of obesity are commonly associated with various environment factors, including demographic, socioeconomic, and behavioral contributions (15). Nevertheless, variation in body weight is largely modulated by a strong genetic component that determines an individual’s susceptibility to these factors. Research conducted through twin and family studies has estimated that obesity has a heritability rate ranging from approximately 40 to 70% (16). Obesity risk prediction is currently a subject of thorough research, with machine learning methods being actively used. Among the commonly used models are logistic regression, naïve Bayes, gradient boosting, random forest, support vector machine, k-nearest neighbor method, as well as various neural network architectures, mainly multilayer perceptron (MLP) and convolutional neural networks (CNN). Majority of the published research relies on non-genetic information, such as social and clinical factors (17–20). Typically, this strategy proves to be fruitful, as it demonstrates a high predictive power. However, it is important to note that the best results are typically achieved when considering both environmental factors and genetic information together. When it comes to polygenic risk prediction for obesity, there are fewer publications, possibly, due to the difficulty of constructing a sufficient dataset containing both genetic and phenotypic information. Nevertheless, machine-learning algorithms have been shown to be accurate and reliable with an average ROC AUC of 0.7 (21, 22). This approach is often used to identify the SNPs that have the most significant impact on obesity (23, 24). It was also demonstrated that age and gender might be among the most important cofactors (23).

Second tested phenotype, type 1 diabetes is an autoimmune disease in which the immune system attacks the cells of the pancreas that produce insulin. Its adverse effects may include high levels of blood sugar, heart disease, stroke, kidney disease, nerve damage, and eye problems. Although nowadays one cannot prevent type 1 diabetes, knowing about the genetic predisposition is important, as early diagnosis and proactive management are key to minimizing the negative effects (25). Moreover, type 1 diabetes is commonly misdiagnosed as type 2 based on clinical indicators (26). Considering that these diseases require different treatment strategies, genetic information becomes of immense importance in classification and predicting type 1 diabetes. While there is an abundance of research concerning type 2 diabetes classification using machine learning approaches on genetic (27, 28) and non-genetic data (29), there is a limited number of publications focusing on type 1 diabetes. Using clinical and socio-economic factors, researchers were able to reach AUC-ROC values up to 0.83 (30, 31). Results that are even more impressive with AUC-ROC of 0.96–0.99 were achieved using metagenomics approach in infants (32, 33). Unfortunately, metagenomics is a rather complex and expensive analysis, and non-genetic classifiers rely on medical history and personal information. Therefore, there is a need for a reliable type 1 diabetes prediction model based on genetic data.

Finally, psoriasis is a chronic autoimmune skin condition that has a strong genetic component. Family and twin studies show strong hereditary patterns, with a higher risk if parents have the condition (34). As GWAS studies show, psoriasis is highly dependent on genetics, polygenic approaches can help to estimate the risks associated with the disease and design a better treatment strategy (35). Heterogeneous type of data is currently being used for psoriasis risk prediction [see (36) for review]. The best results (accuracy up to 98%) of machine learning classification algorithms achieved using gene expression data in affected and healthy cells (37). Unfortunately, such an approach is not suited to early prediction, since it analyzes the affected cells. Using genetic information, it is possible to predict psoriasis before the disease manifests itself in any way.

In this paper, we present our studies on how epistasis complicates a disease classification. For this purpose, we trained machine learning models including deep learning architectures on simulated data containing phenotypes with epistasis of varying complexity. Then we verified our machine learning models on real genetic data collected for three phenotypes: obesity, type 1 diabetes and psoriasis.

## 2 Materials and methods

### 2.1 Epistasis simulation experiments

In order to thoroughly investigate how the contribution of epistasis affects phenotype, as well as to systematically evaluate the performances of various machine learning algorithms for a particular disease, we conducted the following experiments. We generated datasets with varying probabilities of phenotype manifestation (Equations 1, 2). The probability consisted of linear and epistatic portions, and was calculated using the following equation:


(1) $$\begin{array}{c} P\left(Y=1|x_{1},\;x_{2},\ldots ,\;x_{k},\;x_{1}^{e},\;x_{2}^{e},\;x_{3}^{e}\right)=\alpha \cdot P\left(Y=1|x_{1},\;x_{2},\ldots ,\;x_{k}\right)\\ +\left(1-\alpha \right)\cdot P\left(Y=1|x_{1}^{e},\;x_{2}^{e},\;x_{3}^{e}\right) \end{array}$$

where $x_{i}\in \left\{0,1,2\right\}$ and $x_{i}^{e}\in \left\{0,1,2\right\}$ are individual genotypes for certain SNPs associated with linear and epistatic effects respectively, *α* $\in \left[0,1\right]$ is a varying proportion regulating how strong the epistatic effect is. The linear part of the probability equation is described by the equation:


(2) $$P\left(Y=1|x_{1},\;x_{2},\ldots ,\;x_{k}\right)=\frac{\exp\left(\beta_{0}+\beta_{1}\cdot x_{1}+\beta_{2}\cdot x_{2}+\ldots +\beta_{k}\cdot x_{k}\right)}{1+\exp\left(\beta_{0}+\beta_{1}\cdot x_{1}+\beta_{2}\cdot x_{2}+\ldots +\beta_{k}\cdot x_{k}\right)}$$

where coefficients $\beta_{i}$ were sampled from the normal distribution N(0, 0.5).

The probability of 3-loci epistasis $P\left(Y=1|x_{1}^{e},\;x_{2}^{e},\;x_{3}^{e}\right)$ is taken from penetrance tables that were generated using PyTOXO package (38). In this experiment 3 penetrance tables were created for 2-loci and 3 tables for 3-loci epistatic models with heritability of 0.10, 0.25 and 0.50 (the details, including frequencies, can be found in Supplementary Tables 1, 2). We generated genotype profiles consisting of 100 and 1,000 SNPs, including 25 and 100, respectively, that are responsible for linear effect and 2 or 3 SNPs corresponding to 2- and 3-loci epistasis. This way we simulated datasets containing 20,000 and 100,000 people with generated genotypes and the described phenotypes. Genotypes were constructed by randomly assigning SNP to 0, 1 or 2 with frequencies of 0.25, 0.5, and 0.25, respectively. In our setup, we fixed the MAF (Minor Allele Frequency) of the generated genotypes. However, additional simulations (Supplementary Figure 1) have shown that the results of the experiments remain consistent even if we vary the MAF. By varying coefficient *α* from 1 to 0, we created phenotypes with gradually increasing epistasis contribution. Each dataset consisted of 1 genotype and 10 targets with different phenotype compositions. As the phenotypic variance explained by genetic variants varies for different alpha values, we calculated the theoretical AUC value. This value is determined when the ground truth coefficients of the model are used.

To further compare how linear and non-linear models perform in cases of strong epistasis and limited data availability, 30 datasets were generated using the GAMETES 2.1 (39). The simulated phenotypes corresponded to three 2-loci epistasis models: additive, multiplicative and threshold. The same heritability of 0.25 was used. In particular, each machine-learning algorithm was trained and tested on 10 replicates that were created for each epistasis case. Penetrance tables used in this simulation were also generated using PyTOXO package (Supplementary Tables 3–5). Datasets for 20,000 people consisted of 1,000 SNPs, including 2 causal ones, associated with 2-loci epistasis, and 998 non-significant variants. All models were trained and tested on 10 replicate datasets, corresponding to one with the epistasis. The purpose of this experiment was to evaluate model stability by measuring mean and standard deviation of each metric across 10 independent training runs.

### 2.2 Real data for obesity, diabetes and psoriasis

#### 2.2.1 Study cohort

In our study, we analyzed the genetic data of 102,519 individuals from the database of Genotek, the Russian consumer genetics and research company (40). Genotek clients included in our analyses provided informed consent for their data to be used for research purposes. The current research was approved by the Genotek Ethics Committee (protocol №17 “Deep Learning captures the effect of epistasis in multifactorial diseases”) and performed in accordance with the Declaration of Helsinki. Each client was asked to fill the questionnaire about lifestyle, body measurements, and diseases. We used these self-reported data to find individuals with a certain condition.

#### 2.2.2 Genotyping and imputation

DNA extraction and genotyping were performed on saliva samples that were genotyped on Illumina Infinium Global Screening Array v.1-v.3 microarrays (~ 650,000 SNPs). All samples in Genotek cohort were processed in batches (192–768 samples per batch). The GenomeStudio software (Illumina, San Diego, CA) and manually created cluster files that were used to cluster the raw signals and call genotypes. SNPs with a call rate < 0.9 within the batch were removed. We removed individuals with sample call rate < 0.97. Then genotype imputation was performed using HRC and 1,000 Genomes reference panels using Beagle 5.1 (9). Imputed variants with DR2 > 0.7 were kept for the downstream analysis. HIBAG was used to impute star alleles for HLA-DQA1 and HLA-DQB1 genes (41).

#### 2.2.3 SNP selection

For each individual we have genome-wide SNP data. The sample size of our cohort is much less than the number of available features (~8–10 millions of SNPs) that is why we trained and validated our models for the subsets of SNPs known to be related to the considered diseases. To obtain 557 SNPs for obesity we used GWAS summary statistics from GIANT consortium study on European population (42). We used PLINK (43) to perform clumping on those summary statistics using our own genotyping data with the following parameters: LD threshold 0.1, minimum *p*-value for index SNP 0.0001, distance 250 kb, to get the final list of SNPs. For the psoriasis 38 SNPs from (44) were selected. Finally, for type 1 diabetes we used star alleles for HLA-DQA1 and HLA-DQB1 genes and additional non-HLA 48 SNPs from (45). List of selected SNPs can be found in Supplementary Data 1.

#### 2.2.4 Phenotype prediction

Phenotypes were defined for Genotek cohort from the data self-reported by individuals. For obesity, we got 50,168 controls and 8,506 cases (cases included individuals with BMI > = 30, controls – BMI < = 25). Gender and age were included into the model as covariates. For the type 1 diabetes, we received 522 cases. We applied propensity score matching, a technique that is widely used in clinical trials to control for confounding (46–48). For that, each patient was matched with 20 controls with similar age and gender. This resulted in 522 cases and 10,440 controls. A similar procedure was performed for psoriasis matching 7 controls per each of 1,543 cases based on propensity score involving age, gender, smoking status and alcohol consumption. Then Synthetic Minority Over-Sampling Technique (SMOTE) was applied to balance the training subsets by increasing the number of cases.

### 2.3 Training and validation of machine learning algorithms

Multilayer perceptron (MLP), convolutional neural network (CNN) and recurrent neural network (RNN), as well as Lasso regression, random forest and gradient boosting models were assessed using a range of performance metrics including accuracy, F1 score, AUC-PR, recall, precision and AUC-ROC. A similar training and testing process was applied to all models. First, the data was one-hot encoded. Then, all models underwent a cross-validation procedure consisting of 5 cycles, where the data was randomly divided into training and test sets in a 60:40 ratio during each iteration. The final metrics are the averages of the results from these cycles. For neural networks a test set was further split into validation and independent test subsets in equal portions. When training neural networks, we used grid search technique in order to find the optimal hyperparameters. Models were trained for a sufficient number of epochs with loss function and AUC-ROC being recorded. Neural network models were trained using the Adam optimizer with a binary cross-entropy loss function. Learning rate periodically changed from 10^−3^ to 10^−4^ every 10 epochs with utilization of CosineAnnealingLR scheduler. Models were trained for fixed number epochs with AUC-ROC and loss function value recorded on every iteration. Training was interrupted if loss function was increasing for more than 5 consecutive epochs. After training, the model with the best performance on validation set was loaded, and metrics were measured on the independent test dataset.

For gradient boosting we tested three different implementations: LightGBM, XGBoost, and GradientBoostingClassifier form sklearn (Supplementary Figure 2). LightGBM showed the best results on simulated data, so we used it as the default gradient boosting model throughout the study.

Similar MLP architectures were implemented in all experiments. It consisted of multiple fully connected layers, including the input layer with 300 or 3,000 nodes, accounting for 100 or 1,000 one-hot encoded SNPs, three hidden layers and a single output node. The number of neurons in the hidden layers was optimized using grid search technique and varied from 300 to 2,500 in the first layer, from 100 to 500 in the second and from 10 to 75 in the last layer. The Rectified Linear Unit (ReLU) activation function was applied to all neurons, except for the output, where a sigmoid activation function was used. Batch normalization was incorporated after each hidden layer to improve training stability and speed. To prevent overfitting, dropout regularization was also used after each layer, except for the output. Hyperparameters that were optimized during the grid search included composition of hidden layers, batch normalization momentum, dropout value after the initial layer and the dropout of other hidden layers. The CNN architecture consisted of two 1D convolution and one fully connected hidden layer. After each fully connected layer ReLU activation function was used, followed by dropout regularization. Number of channels, kernel size, stride and the number of neurons in the fully connected layers varied during the hyperparameter optimization. In particular, the number of channels changed from 150 to 1,000, different kernel and stride values were tested ranging from 1 to 4, and fully connected layers varied from 50 to 100. The RNN architecture consisted of two components: one LSTM (Long Short-Term Memory) and one fully connected output layer, separated by a dropout layer. The dimensionality of the LSTM hidden state, as well as the dropout value were optimized, and changed from 50 to 300 and from 0.7 to 0.9, respectively. Finally, in the experiments with real data, a combination of RNN-CNN networks was also tested. It consisted of one LSTM, two convolution, one fully connected hidden layer. Similar to the previous architectures, ReLU activation function and dropout layer were used after the hidden layer, while the output layer was followed by a sigmoid activation function. Hyper-parameters and their values were similar to the setup described above.

## 3 Results

**Figure 1:**
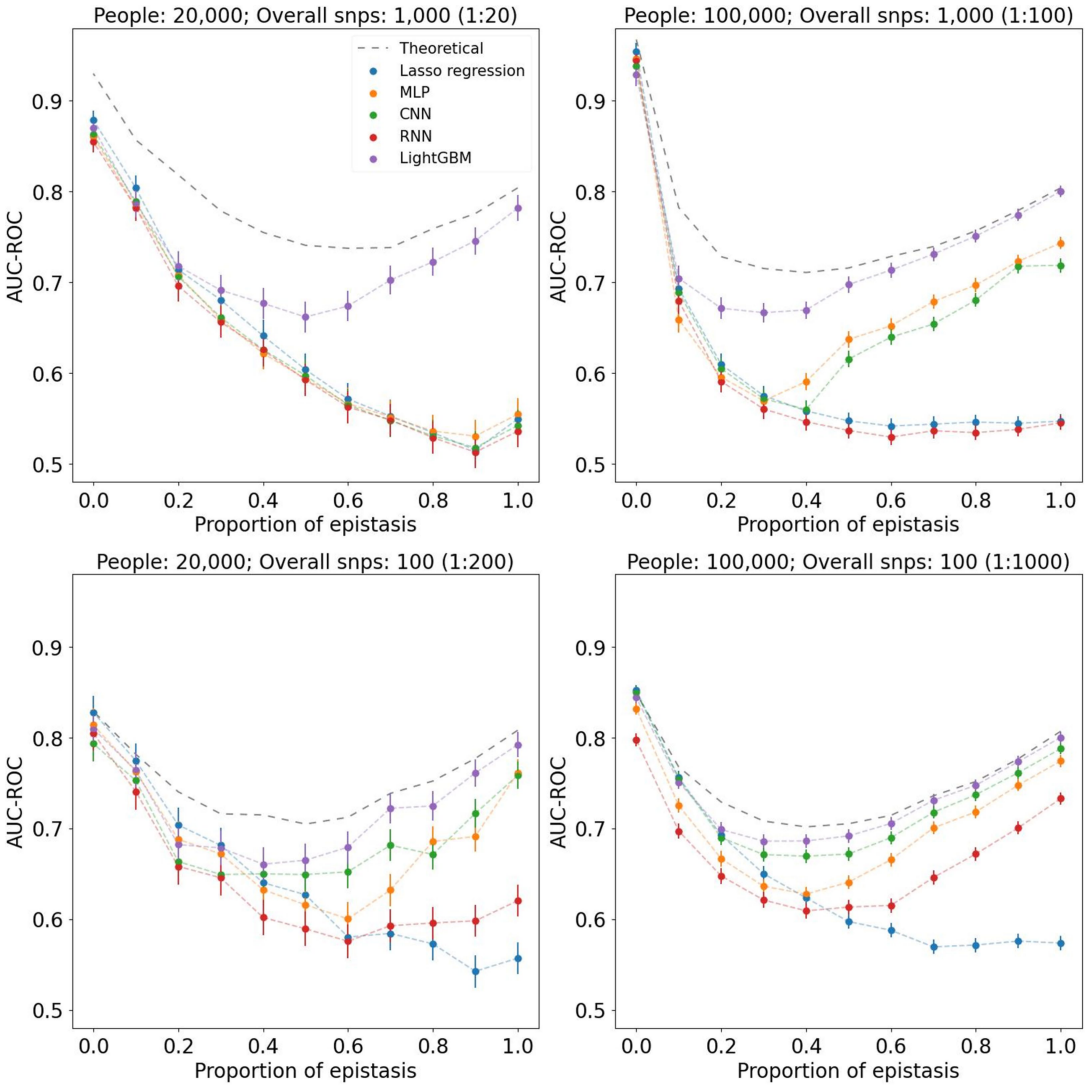
Graphs of AUC-ROC values measured for different machine learning methods. Each value at the X axis corresponds to a phenotype with a certain contribution of epistasis. For each AUC-ROC value, the boundaries of the 95% confidence interval are indicated. Each graph corresponds to a different dataset composition and feature-to-instance ratio. In all cases, 3-loci epistasis model with heritability of 0.25 was used.

To assess the performance of different machine learning models we first tested them on simulated datasets with varying epistasis effects. The phenotypes we used differed both in the models of epistasis and in the strength of its contribution. We used 2- and 3-loci epistasis with heritabilities of 0.1, 0.25 and 0.5, resulting in six different models. The contribution of epistasis to the probability of phenotype manifestation for each model varied from 0 to 1. We used cohorts of 20,000 and 100,000 people with approximately equal case/control ratio. The results are summarized in Figure 1. It is important to note that each coefficient within the combined linear and epistasis model yields a distinct model, each explaining a different proportion of the phenotypic variance. Consequently, each coefficient is associated with its own theoretical value for AUC. In all scenarios, models behave similarly in the far-left side of the graphs that correspond to low epistasis contribution. From approximately 0.3 ratio, where epistasis effect approaches 30%, we start seeing the noticeable difference between linear and non-linear models. Thus, Lasso regression results decline as the ratio increases, while gradient boosting and neural networks demonstrate the ability to capture epistasis, as their metrics grow in the far-right side of the graphs. It should be noted that for the best performance neural networks require an extensive dataset with high feature-to-instance ratio, which depends on complexity and quality of data. For instance, when we used 20,000 instances for 1,000 features (1:20 ratio), none of the neural networks was able to distinguish strong epistasis phenotypes. That ratio is also the case when all metrics were the furthest from the theoretical AUC-ROC. As the feature-to-instance ratio increases, models stabilize their performance. When we used 10,000 instances with 100 features, all models showed their most stable results, nearly reaching the theoretical AUC-ROC. While MLP and CNN demonstrated similar performances, RNN had the worst metrics and least stability among all non-linear models. Additionally, we tested how heritability of 2-loci and 3-loci epistatsis affects the complexity of phenotype profile (see Supplementary Figure 3). The simulation indicates that the higher-order epistasis is harder to detect.

Manifestations of epistasis take quite complex forms. To better understand how different forms of epistasis may affect the predictive abilities of machine-learning algorithms, we simulated three theoretical epistasis models previously described by Marchini et al. (49): additive (also known as “Multiplicative within and between loci”), multiplicative and threshold. First model describes an interaction within and between loci, where effect is proportional to the number of causal alleles. The second type is called multiplicative, and it is characterized by constant probability of a disease, unless both loci have at least one causal allele, in which case the effect grows similarly to the additive model. Finally, the threshold model manifests as two types of constant effects: minor and major effect, where the latest corresponds to both loci possessing disease-associated alleles (refer to Figure 1 of Marchini et al. (49) for graphical presentation of the models). In this simulation, we tested sub-optimal conditions for training models using datasets with low feature-to-instance ratio (1:20).

The results obtained on simulated data convincingly prove that usage of non-linear models might be beneficial in predicting the risks heavily influenced by epistasis (Table 1).

**Table 1:** Comparison of metrics produced by tested machine learning models on simulated data with three types of epistasis.

	AUC-ROC	Accuracy	Recall	Precision	F_1_-score
Additive					
Lasso regression	0.850 ± 0.001	0.770 ± 0.001	0.768 ± 0.002	0.772 ± 0.001	0.770 ± 0.001
MLP	0.846 ± 0.001	0.769 ± 0.002	0.727 ± 0.011	0.794 ± 0.005	0.758 ± 0.004
CNN	0.843 ± 0.003	0.725 ± 0.021	0.842 ± 0.026	0.699 ± 0.025	0.756 ± 0.008
RNN	0.854 ± 0.001	0.773 ± 0.001	0.753 ± 0.003	0.785 ± 0.002	0.768 ± 0.002
Random Forest	0.854 ± 0.001	0.775 ± 0.001	0.760 ± 0.003	0.784 ± 0.002	0.772 ± 0.001
Gradient Boosting	**0.877 ± 0.001**	0.805 ± 0.002	0.775 ± 0.003	0.781 ± 0.002	0.814 ± 0.00
Multiplicative					
Lasso regression	0.721 ± 0.002	0.662 ± 0.002	0.640 ± 0.003	0.670 ± 0.002	0.655 ± 0.002
MLP	0.733 ± 0.002	0.646 ± 0.014	0.719 ± 0.030	0.642 ± 0.022	0.670 ± 0.003
CNN	0.686 ± 0.022	0.593 ± 0.026	0.800 ± 0.058	0.597 ± 0.030	0.661 ± 0.003
RNN	0.730 ± 0.002	0.674 ± 0.002	0.625 ± 0.003	0.693 ± 0.002	0.658 ± 0.002
Random Forest	0.734 ± 0.002	0.668 ± 0.002	0.681 ± 0.003	0.664 ± 0.003	0.672 ± 0.002
Gradient Boosting	**0.771 ± 0.002**	0.731 ± 0.002	0.659 ± 0.002	0.769 ± 0.002	0.710 ± 0.002
Threshold					
Lasso regression	0.654 ± 0.002	0.608 ± 0.002	0.611 ± 0.003	0.607 ± 0.002	0.609 ± 0.002
MLP	0.687 ± 0.002	0.591 ± 0.008	0.717 ± 0.025	0.577 ± 0.010	0.636 ± 0.004
CNN	0.662 ± 0.009	0.556 ± 0.017	0.673 ± 0.106	0.529 ± 0.068	0.553 ± 0.066
RNN	0.672 ± 0.002	0.621 ± 0.001	0.578 ± 0.004	0.633 ± 0.002	0.604 ± 0.002
Random Forest	0.680 ± 0.003	0.611 ± 0.002	0.650 ± 0.004	0.603 ± 0.002	0.626 ± 0.002
Gradient Boosting	**0.695 ± 0.001**	0.656 ± 0.001	0.507 ± 0.002	0.723 ± 0.002	0.596 ± 0.001

Indicated values are mean and standard deviation measured across 10 replicate datasets for each epistasis type. The best performance metrics are indicated in bold.

**Figure 2:**
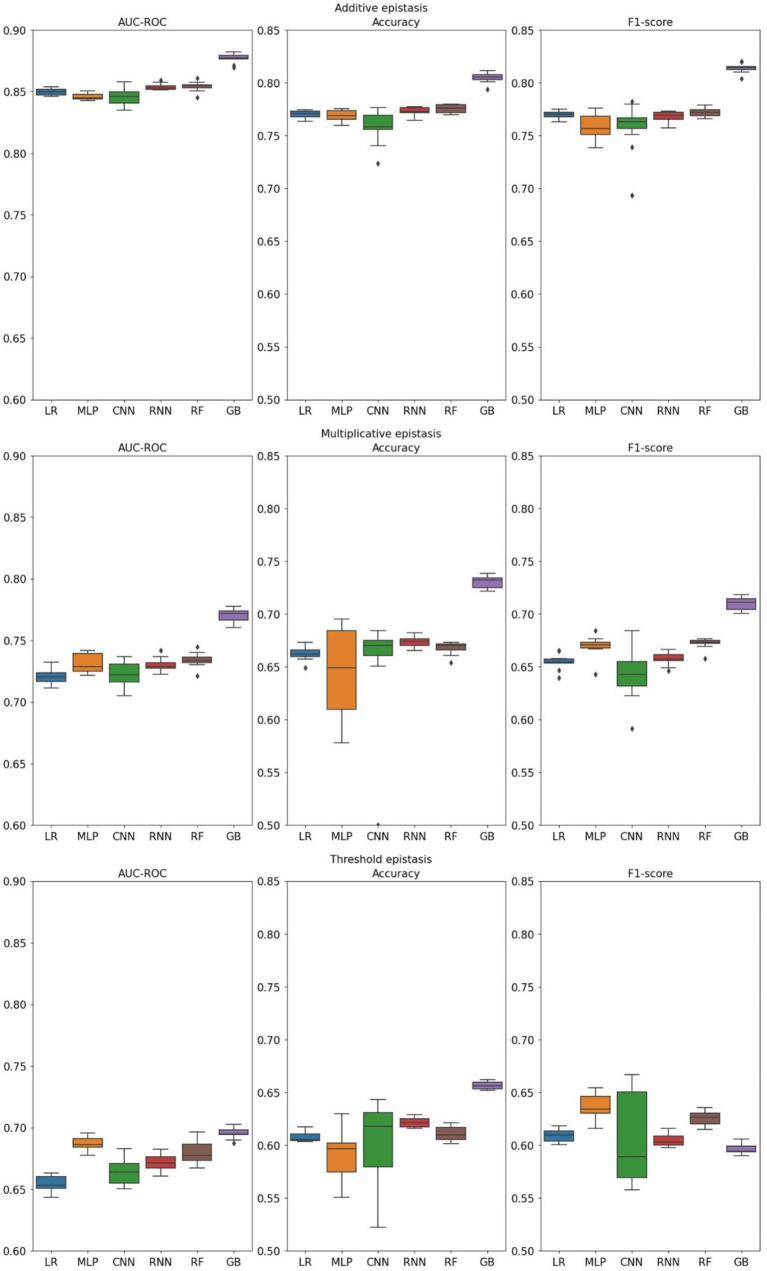
Distributions of metrics measured for different machine learning models. Three theoretical forms of epistasis and their corresponding datasets were generated using GAMETES (LR, Lasso regression; GB, Gradient Boosting).

Additive epistasis (Figure 2), being the simplest form, is relatively easy to detect because its penetrance table contains only explicit dependence on the alleles. Indeed, that is proven by the simulation results, as both linear and non-linear models performed at a similar level, with LightGBM outperforming the others. When classifying multiplicative epistasis, all performance metrics reduced significantly. While all non-linear models, except for CNN, demonstrated higher AUC-ROC, the best results were achieved by the gradient boosting (0.771 ± 0.002). Noticeably, MLP and CNN showed the highest variance across all metrics. Finally, the threshold model corresponded to the lowest values across all metrics. Highest AUC-ROC was achieved by the gradient boosting (0.695 ± 0.001), followed by MLP (0.687 ± 0.002) and RF (0.680 ± 0.003). The worst results were demonstrated by the linear model (Lasso regression) (0.654 ± 0.002). For the reasons that will be discussed in the next section, accuracy and F1-score were found to be in reverse correlation. For instance, in the threshold model of epistasis simulation MLP had the highest F1-score and the lowest accuracy. This correlation stays true for all models. Here we also tested three gradient boosting algorithms and comparison of different implementations of gradient boosting algorithm performances can be found in Supplementary Figure 2.

The results of the models for classification of real phenotypes based on genetic data are presented in Table 2. For obesity, all tested models performed similarly. Among neural network architectures only MLP marginally surpasses Lasso regression, while CNN, RNN, hybrid RNN-CNN and other gradient boosting models stay behind. For type 1 diabetes, models based on RNN appeared to be the most effective according to average metrics, closely followed by the hybrid RNN-CNN and MLP. Finally, in experiments with psoriasis data, all tested models demonstrated similar results.

**Table 2:** Average metrics of various machine learning models for real genetic data.

	Obesity		Type 1 diabetes		Psoriasis	
	AUC-ROC	Accuracy	AUC-ROC	Accuracy	AUC-ROC	Accuracy
Lasso regression	0.773 ± 0.002	0.857 ± 0.001	0.787 ± 0.004	0.759 ± 0.004	0.699 ± 0.004	0.696 ± 0.004
MLP	**0.775 ± 0.002**	0.855 ± 0.001	0.820 ± 0.004	0.764 ± 0.021	0.693 ± 0.004	0.641 ± 0.006
CNN	0.769 ± 0.002	0.849 ± 0.001	0.815 ± 0.003	0.741 **±** 0.027	0.700 ± 0.003	0.584 ± 0.012
RNN	0.752 ± 0.002	0.851 ± 0.001	**0.823 ± 0.004**	0.798 ± 0.006	0.697 ± 0.003	0.679 ± 0.005
RNN-CNN	0.763 ± 0.002	0.854 ± 0.001	0.822 ± 0.004	0.747 **±** 0.010	**0.701 ± 0.004**	0.648 ± 0.011
Gradient Boosting	0.774 ± 0.002	**0.858 ± 0.001**	0.805 ± 0.005	**0.954 ± 0.001**	0.688 ± 0.004	**0.869 ± 0.001**
Random Forest	0.714 ± 0.001	0.855 ± 0.001	0.792 ± 0.006	**0.954 ± 0.001**	0.676 ± 0.004	**0.869 ± 0.001**

The targeted phenotypes include obesity, type 1 diabetes and psoriasis. The results represent the average value and the standard deviation of the measured metrics on 10 independent training and testing cycles. The best performance metrics are indicated in bold.

## 4 Discussion

In the simulation experiment with varying strength of epistasis, we clearly see the difference in linear and non-linear model performances. When phenotype consists of only linear effects, all models provide nearly identical results. Once the epistasis vs. linear proportion reaches 30–40%, we see how performance of Lasso regression started to decline, while non-linear approaches retain their prediction ability. Moreover, as the linear dependence decreases, these models produce higher performance metrics. This experiment proves that in certain phenotypes non-linear algorithms might be the tool of choice. However, in the absence of or with insignificant effect of epistasis the linear models can compete with non-linear. This may be the reason that in a number of publications linear models or classical PRS algorithms provide similar (50) or even better results (51) than non-linear. In addition, the availability of quality data for training and testing plays a key role in the resulting outcome. Thus, when the feature-to-instance ratio is low (for example, 1:20), none of the neural networks was able to get sufficient training. Even when the ratio grew to 1:200, RNN was still unable to detect epistatic phenotypes. Only with the highest ratio of 1:1000, all non-linear models provided satisfactory results. Finally, the complexity and order of epistasis, which can consist of 2, 3, or more loci, may vary, leading to varying levels of inability to detect epistasis by linear models. Figure 1 summarizes the results for 3-loci epistasis with heritability of 0.25, where AUC-ROC of Lasso regression drops consistently regardless of the feature-to-instance ratio. This is not always the case. For instance, 2-loci epistasis with extremely high heritability of 0.5, is detected by Lasso regression with nearly the same metrics as the other models. Higher order epistasis, such as 3-loci, is much harder to detect by linear models, even when it has large heritability. Supplementary Figure 3 provides a summary of performances for 2- and 3-loci epistasis with heritability of 0.1, 0.25 and 0.5.

Models showed expected results for additive epistasis, which is the simplest form, and its marginal effects are easy to estimate. All metrics were similar with the exception of Gradient Boosting which demonstrated the best results in all three epistasis types. Essentially, additive form demonstrates linear behavior, and therefore any model can produce satisfying results. On the other hand, more complex types of epistasis can pose a problem. Thus, the most challenging aspect of the multiplicative epistasis is the abrupt switch from constant to additive marginal effects when both alleles include causal variants. Non-linear models can distinguish such complex phenotypes, while linear cannot produce the same results. This tendency is even more obvious when it comes to the threshold type that has only two constant marginal effects. It seems that Lasso regression can comprehend the additive part of multiplicative epistasis; therefore, when both loci possess causal alleles, it classifies the cases similarly to non-linear competitors. However, threshold type abruptly switches the marginal effects from one constant value to another. That behavior is a serious problem for a linear model, which becomes apparent when we compare AUC-ROC of Lasso regression and the other models.

Special attention should be paid to the trade-off between accuracy and F1-score. By changing the threshold of a classification probability, we can balance these metrics. In fact, the accuracy measured in these experiments is reversely correlated with F1-score. For example, in threshold epistasis, MLP had the highest F1-score and the lowest accuracy. Graphs demonstrating this behavior are presented in Supplementary Figure 4. Thus, by choosing an appropriate probability threshold, it is possible to increase one of two metrics at the expense of the other.

We have conducted a simulation study that has several limitations. One of these limitations is the number of SNPs in the synthetic dataset. Deep learning models are time- and resource-intensive, so to make the computations feasible, we limited the number of simulated SNPs to 1,000. On one hand, this might be reasonable if we use GWAS-based filtration to select the top SNPs for further analysis. On the other hand, we believe that in the near future, deep learning methods will become faster and more cost-effective. Additionally, we fixed the MAF in these simulations. More extensive and realistic simulations could be applied in future research to study the feasibility of epistasis detection in greater detail.

In this study, we assumed that genotype data is available for a large cohort. This assumption is quite reasonable given the increasing number of biobanks and genetic testing companies with hundreds of thousands of genomes that have emerged in recent years. However, researchers often use GWAS summary statistics and additional cohorts to train and validate polygenic risk scores (PRS; e.g., PRSice 2, LDPred2, lassosum, etc.). Some methods, like LDPred2, recalculate the weights to account for linkage disequilibrium (LD). As a direction for future research, it would be interesting to test the ability of these methods to account for epistasis using both simulated and real data.

Overall, the experiments on simulated data have shown that nonlinear models can outperform conventional linear approaches for some phenotypes. The difference in performance considerably increases with the increase of contribution of epistasis.

The results obtained on the simulated data were confirmed by the experiments on the real genetic data, and are in agreement with previously published studies (4). Thus, for obesity, all models demonstrated similar results. The explanation why non-linear approaches do not show a substantial difference from the linear model may lie in the nature of this disease. Even though, a number of epistatic interactions may affect body mass index (52), GWAS statistics revealed nearly a thousand of SNPs highly associated with the BMI (with *p*-values <1 × 10^−8^) (53). Therefore, it is possible that the impact of these interactions can be overshadowed by SNPs with linear contribution to phenotype. Furthermore, obesity is highly correlated with various socio-economic factors. Thus, a person may be genetically predisposed to obesity, but lifestyle and easy availability of high-calorie food remain the key risk factors (13, 15).

The results for type 1 diabetes support the idea that some phenotypes may benefit from usage of non-linear models. It has been proven that diabetes is highly affected by epistatic interactions (37). Moreover, typical GWAS for type 1 diabetes consists of only a small number of SNPs with statistically significant associations (54). Thus, the combination of a small number of genetic features, some of which are associated with strong epistasis, makes diabetes an excellent example of a disease for which non-linear models will be more reliable in genetic risk prediction. In fact, all tested neural network architectures demonstrated high and consistent predictive capabilities, while gradient boosting and random forest remained at the level of performance of Lasso regression.

Although a number of studies shows that epistasis plays a significant role in the manifestation of psoriasis (55, 56), tested non-linear approaches were not able to outperform linear models. One possible reason may lie in the insufficient number of cases available for training. It is known that in order for advanced models to reach their full potential, it is necessary to provide an extensive dataset with balanced data. Another possible explanation is that the effect of epistasis could be less than that for the type 1 diabetes. As it was shown on simulated data, non-linear methods are most effective for a substantial epistatic interaction. Finally, phenotypic information is self-reported and was obtained from client’s voluntary survey. Since some clients might not want to disclose information about their illnesses, some of the data may have been incorrectly marked up. Thus, some of the controls could actually relate to cases, thereby creating an error in the dataset. However, it is important to note that the metrics obtained in our study are comparable to the metrics from studies of other clinical cohorts (57, 58).

The models evaluated in this study are not designed for epistasis detection but rather for predicting phenotypes based on genetic data. The most significant increase in AUC was observed for Type 1 Diabetes, with RNN achieving an AUC of 0.82 compared to 0.79 for Lasso, potentially indicating the presence of epistatic effects. Conversely, non-linear models yielded the same AUC for obesity and psoriasis. However, this should not be interpreted as evidence of the absence of epistasis for these two phenotypes, as our study has several limitations. For instance, epistatic genetic variants may be excluded during the pre-selection of SNPs, and our sample size may be insufficient to detect weak epistatic interactions. From a practical standpoint, our findings corroborate previous research suggesting that non-linear models for Type 1 Diabetes outperform linear models and are more suitable for individual risk estimation.

Experiments on simulated data have shown that the results of various machine learning models directly depend on the complexity of targeted phenotype. Thus, all models show similar results when dependence between SNPs and disease is linear. In the case when epistasis plays a significant role, non-linear models significantly outperform linear. For different models of epistasis, whether it is additive, multiplicative, or threshold, the performance of different machine-learning models directly depends on the order and complexity of epistasis. Another crucial factor in model performance is the feature-to-instance ratio, since complex non-linear approaches, especially neural networks, require large balanced datasets. Overall, we created two simulation setups to assess different aspects of epistasis: its form and strength. Clearly, these experiments cannot fully capture the complex nature of a multifactorial disease. However, such analysis of separate characteristics of epistasis in isolated experiments allows us to better understand this intricate phenomenon. Creating a simulation that more closely resembles a real multifactorial disease will be one of the tasks in our future studies.

Experiments with real genetic data further support the thesis that non-linear models outperform linear approaches, especially for phenotypes with significant contribution of epistasis. Thus, non-linear models outperform linear in type 1 diabetes that, according to recent studies, has a significant contribution of epistasis and a small number of causal SNPs. For obesity, gradient boosting model was able to slightly improve prediction performance of linear models, though the total amount of SNPs that have genetic contribution to this phenotype is highly disputable and the effect of epistasis is smaller. It is worth mentioning that according to our simulation results, non-linear methods perform similarly to linear models when epistasis takes a simple form or has a small effect. Unfortunately, it is impossible to draw an unambiguous conclusion about the characteristics of epistasis in a real disease based only on statistical analysis and machine learning. Meanwhile, a major advantage of non-linear methods is that, unlike their linear alternatives, they do not require an accurate account of intricate genetic interactions for high performance.

Finally, it is worth noting that there is no single model that can effectively predict the risks of several diseases at the same time. Different diseases have different risk factors, including varying complexity of the epistatic contribution. Therefore, for each disease it is necessary to build the optimal model, which, most likely, will not suit another phenotype.

## Data Availability

The data analyzed in this study is subject to the following licenses/restrictions: simulated datasets, simulation and training/testing code, as well as deep learning modes are available on Github (https://github.com/DLepistasis/Deep-Learning-captures-the-effect-of-epistasis-in-multifactorial-diseases). Patients’ data cannot be disclosed for privacy reasons. Requests to access these datasets should be directed to Alexander Rakitko, rakitko@genotek.ru.
