# Supplementary material for: Deep learning captures the effect of epistasis in multifactorial diseases
Source: Front Med (Lausanne). 2025 Jan 7;11:1479717. doi: 10.3389/fmed.2024.1479717 (PMC11746092; doi:10.3389/fmed.2024.1479717)
Supplement: Supplementary file 1 [file Data_Sheet_1.pdf]

Deep Learning captures the effect of epistasis in multifactorial diseases

Vladislav Perelygin[1], Alexey Kamelin[1,2], Nikita Syzrantsev[2], Layal Shaheen[2,3], Anna Kim[2], Nikolay Plotnikov[2], Anna Ilinskaya[4], Valery Ilinsky[4], Alexander Rakitko[1,2,*], Maria Poptsova[1,*]

[1]International Laboratory of Bioinformatics, AI and Digital Sciences Institute, Faculty of Computer Science, HSE University, Moscow, Russia

[2]Genotek Ltd., Moscow, Russia

[3]Phystech school of Biological and Medical Physics, Moscow Institute of Physics and Technology, Moscow, Russia

[4]Eligens SIA, Mārupe, Latvia

*** Correspondence:**
Alexander Rakitko, Maria Poptsova
rakitko@genotek.ru, mpoptsova@hse.ru

Supplementary Material

Supplementary Table 1. Disease likelihood in additive, multiplicative and threshold types of epistasis. These schemes where supplied to PyTOXO to generate penetrance tables that were later used in GAMETES.

Genotype	Additive	Multiplicative	Threshold
AABB	x	x	x
AABb	$x*(1+y)$	x	x
AAbb	$x*(1+y)^2$	x	x
AaBB	$x*(1+y)$	x	x
AaBb	$x*(1+y)^2$	$x*(1+y)$	$x*(1+y)$
Aabb	$x*(1+y)^3$	$x*(1+y)^2$	$x*(1+y)$
aaBB	$x*(1+y)^2$	x	x
aaBb	$x*(1+y)^3$	$x*(1+y)^2$	$x*(1+y)$
aabb	$x*(1+y)^4$	$x*(1+y)^4$	$x*(1+y)$

Supplementary Table 2. Penetrance table of 3-loci epistasis with heritability of 0.25 that was used in simulation with varying epistatic contribution.

	SNP 2 (MAF = 0.25)					
SNP 1 (MAF = 0.25)	**Genotype**	**BB**	**Bb**	**bb**	**Marginal penetrance**	SNP 3 (MAF = 0.25), CC
	**AA**	0.643	0.258	0.542	0.492	
	**Aa**	0.281	0.812	0.473	0.492	
	**aa**	0.404	0.68	0.165	0.492	
	**Marginal penetrance**	0.492	0.492	0.492		
	**SNP 2 (MAF = 0.25)**					
SNP 1 (MAF = 0.25)	**Genotype**	**BB**	**Bb**	**bb**	**Marginal penetrance**	SNP 3 (MAF = 0.25), Cc
	**AA**	0.264	0.845	0.432	0.492	
	**Aa**	0.81	0.014	0.501	0.492	
	**aa**	0.64	0.188	0.986	0.492	
	**Marginal penetrance**	0.492	0.492	0.492		
	**SNP 2 (MAF = 0.25)**					
SNP 1 (MAF = 0.25)	**Genotype**	**BB**	**Bb**	**bb**	**Marginal penetrance**	SNP 3 (MAF = 0.25), cc
	**AA**	0.509	0.481	0.411	0.492	
	**Aa**	0.482	0.487	0.617	0.492	
	**aa**	0.404	0.627	0.481	0.492	
	**Marginal penetrance**	0.492	0.492	0.492		

Supplementary Table 3. Penetrance table of the additive epistasis model.

	SNP 2 (MAF = 0.25)				
SNP 1 (MAF = 0.25)	**Genotype**	**BB**	**Bb**	**bb**	**Marginal penetrance**
	**AA**	0.001	0.006	0.033	0.005
	**Aa**	0.006	0.033	0.181	0.027
	**aa**	0.033	0.181	1	0.149
	**Marginal penetrance**	0.005	0.027	0.149	

Supplemental Table 4. Penetrance table of the multiplicative epistasis model.

	SNP 2 (MAF = 0.25)				
SNP 1 (MAF = 0.25)	**Genotype**	**BB**	**Bb**	**bb**	**Marginal penetrance**
	**AA**	0.007	0.007	0.007	0.007
	**Aa**	0.007	0.025	0.085	0.019
	**aa**	0.007	0.085	1	0.099
	**Marginal penetrance**	0.007	0.019	0.099	

Supplementary Table 5. Penetrance table of the multiplicative epistasis model.

	SNP 2 (MAF = 0.25)				
SNP 1 (MAF = 0.25)	**Genotype**	**BB**	**Bb**	**bb**	**Marginal penetrance**
	**AA**	0.377	0.377	0.377	0.377
	**Aa**	0.377	1	1	0.650
	**aa**	0.377	1	1	0.650
	**Marginal penetrance**	0.377	0.650	0.650	

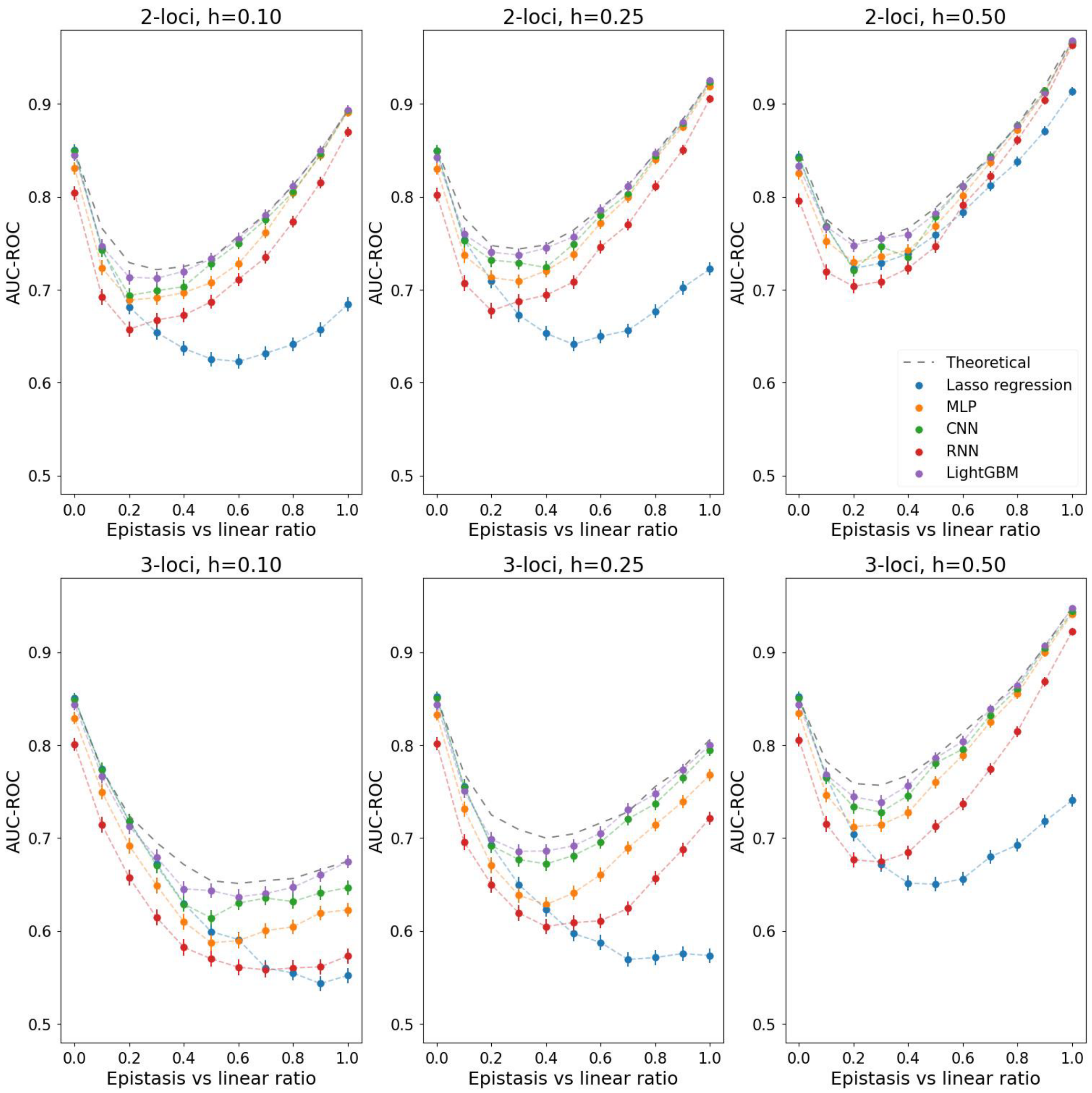

Supplementary Figure 1. AUC-ROC values measured for different machine learning methods on simulated epistatic data. Simulation included 100,000 people with 100 SNPs, providing feature-to-instance ratio of 1:1,000. Each value on the abscissa axis corresponds to a phenotype with a certain contribution of epistasis (coefficient α). For each AUC-ROC value, the boundaries of the 95% confidence interval are indicated. Each figure corresponds to one of the epistasis models: 2- or 3-locus model with heritability equal to 0.1, 0.25 or 0.5.

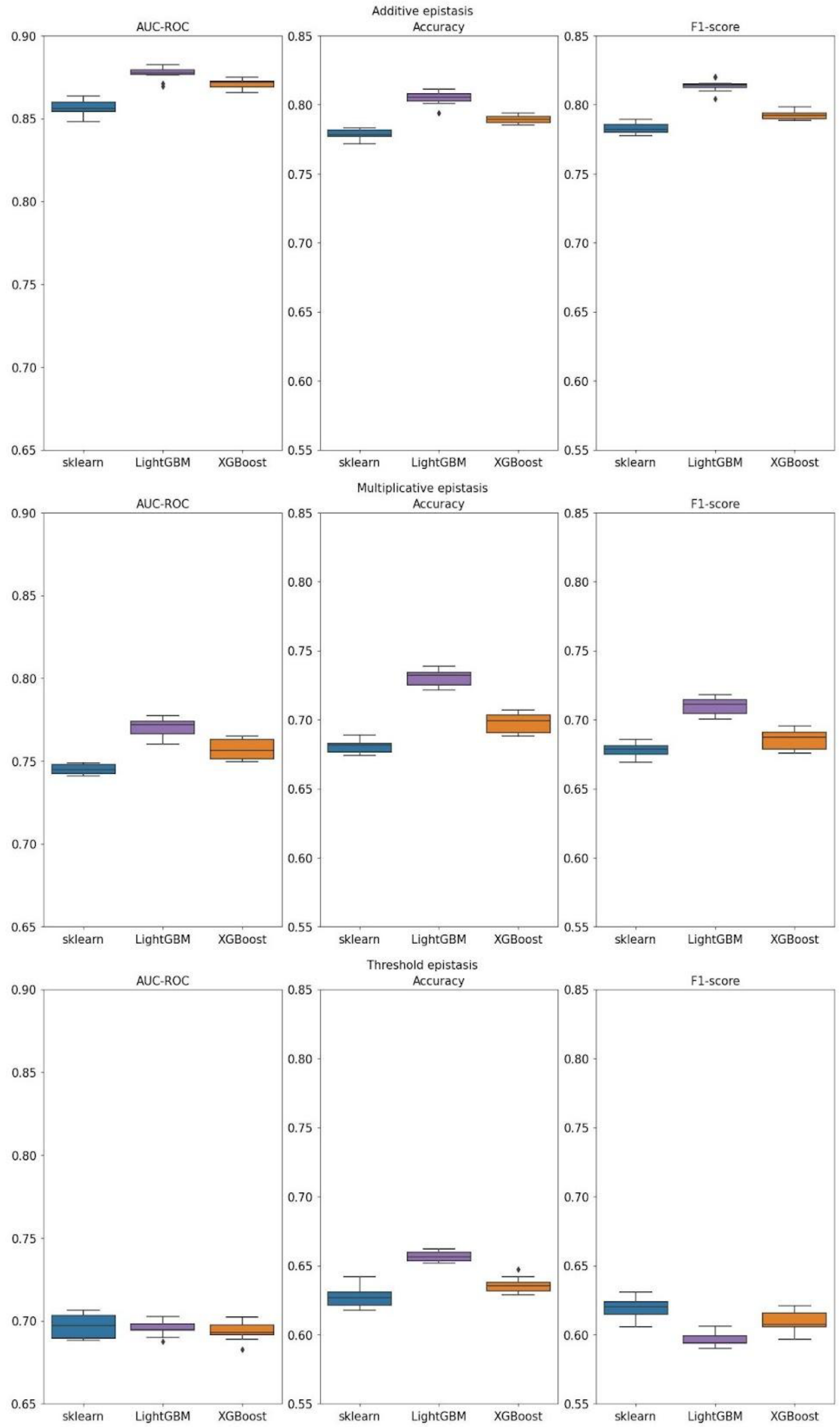

Supplementary Figure 2. Performance comparison of three variants of gradient boosting that were tested in this study: gradient boosting from sklearn library, LightGBM and XGBoost. Data corresponds to three types of simulated epistasis: additive, multiplicative and threshold.

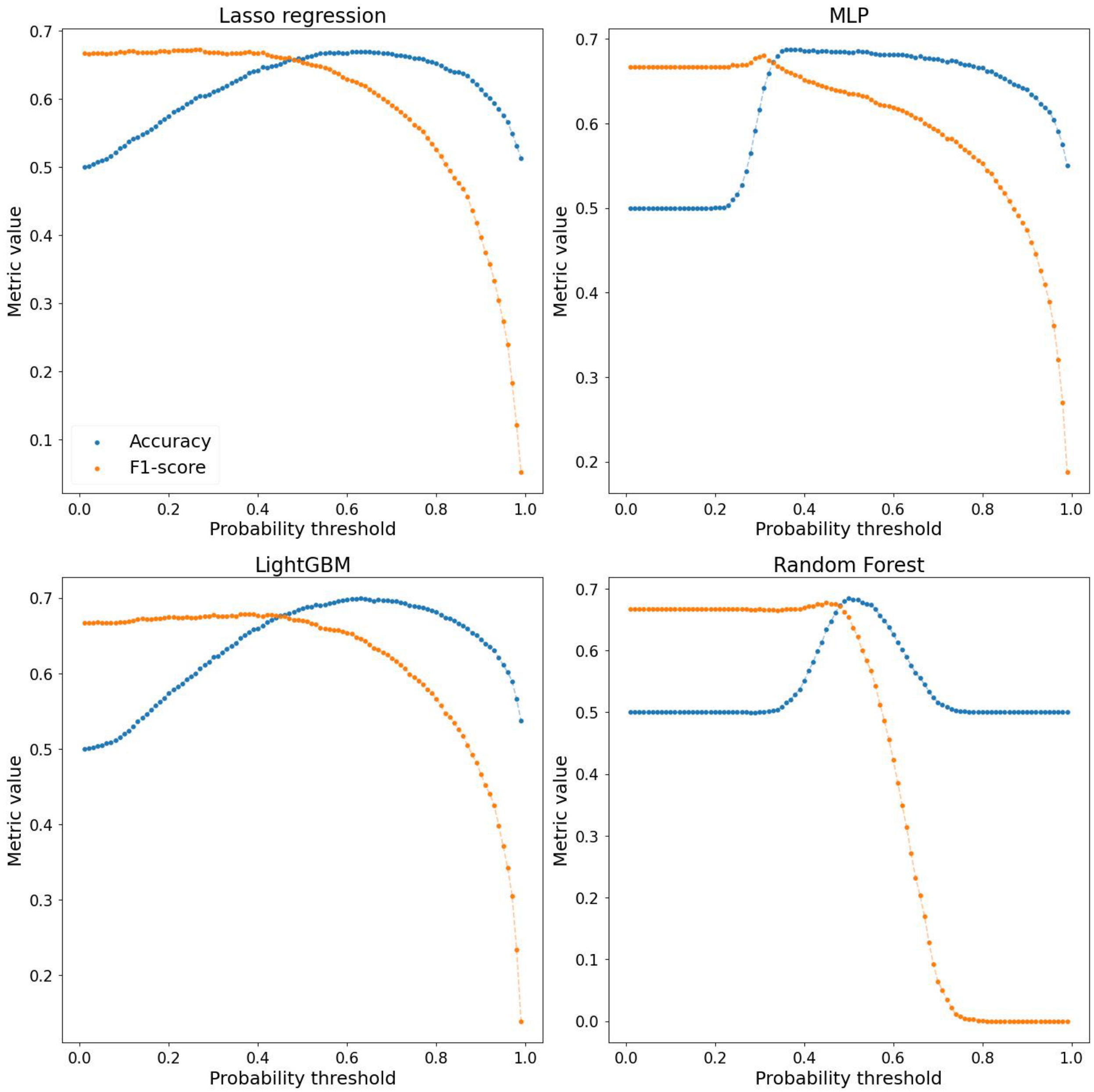

Supplemental Figure 3. Metrics trade-off for different machine leering models on simulated dataset representing multiplicative epistasis. The X-axis indicates the values by which the result of model prediction is attributed to a disease.

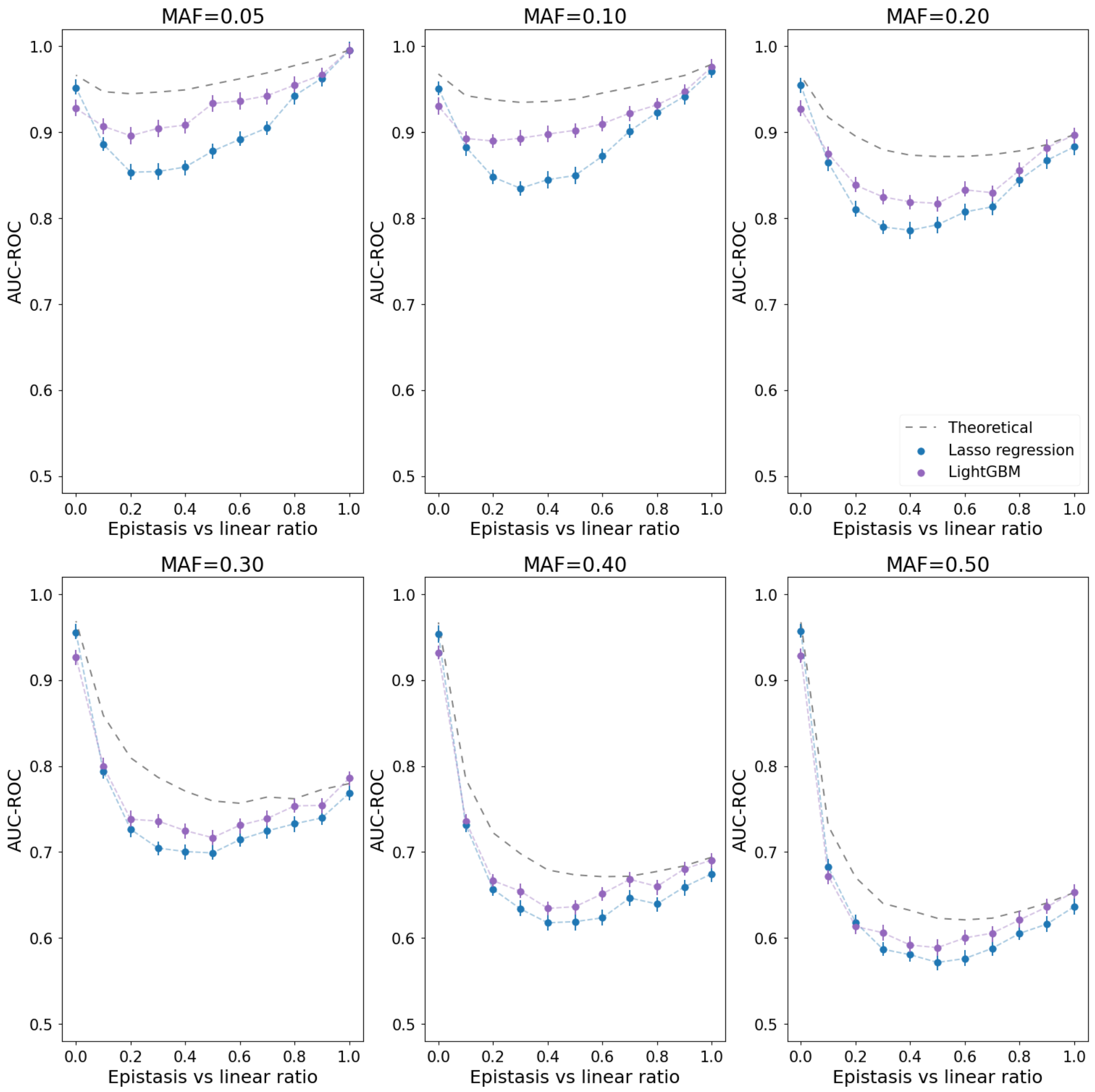

Supplementary Figure 4. AUC-ROC values measured for different machine learning methods on simulated epistatic data with different MAF frequency. Simulation included 100,000 people with 100 SNPs, providing feature-to-instance ratio of 1:1,000. Each value on the abscissa axis corresponds to a phenotype with a certain contribution of epistasis (coefficient α). For each AUC-ROC value, the boundaries of the 95% confidence interval are indicated..
